# Angiotensin Converting Enzyme Inhibitors (ACEI) and doxorubicin pharmacokinetics in women receiving adjuvant breast cancer treatment

**DOI:** 10.1186/s40064-015-0802-4

**Published:** 2015-01-23

**Authors:** Anne Blaes, Daniel Duprez, Todd Defor, Ryan Shanley, Heather Beckwith, Tufia Haddad, David Potter, Douglas Yee, Kinjal Sanghavi, Pamala Jacobson

**Affiliations:** Division of Hematology/Oncology/Transplantation, University of Minnesota, 420 Delaware Street, S.E., MMC 480, Minneapolis, MN 55455 USA; University of Minnesota, Division of Cardiology, Minneapolis, MN USA; Biostatistics Core, University of Minnesota, Minneapolis, MN USA; Mayo Clinic, Rochester, MN USA; College of Pharmacy, University of Minnesota, Minneapolis, MN USA

**Keywords:** Doxorubicin, Angiotensin Converting Enzyme Inhibitors, Pharmacokinetics, Cardioprotection, Drug interaction, Enalapril, Breast cancer

## Abstract

**Purpose:**

Doxorubicin (DOX) chemotherapy can cause cardiac complications. Angiotensin converting enzyme inhibitors (ACEI) may protect against these complications. We performed a pharmacokinetics (PK) study to determine whether DOX levels are altered in the presence of ACEI.

**Methods:**

In this randomized, cross-over, single-blinded drug-drug interaction study, 19 women with breast cancer prescribed DOX and cyclophosphamide every 14 days received one cycle of DOX chemotherapy with ACEI enalapril 10 mg daily and another cycle of DOX with placebo. Blood samples for DOX and doxorubicinol were drawn at baseline, 0.5, 1.0, 2.0, 4.0, 24.0 and 48.0 hours after infusion with and without ACEI enalapril. Correlative laboratories were also obtained. PK data was analyzed using non compartmental methods and DOX and doxorubicinol area under the curve (AUC) 0 to infinity, Cmax and half-life were estimated. Paired t-tests were used to determine whether DOX and its metabolite were altered with the use of enalapril (P < 0.05).

**Results:**

17 women (median age 45 years) received 60 mg/m2 DOX every two weeks for four cycles. Mean (SD) AUC0- ∞ for DOX and doxorubicinol with enalapril exposure was 1185.56 (44.64) hr*ng/ml and 1040 (80.6) hr*ng/ml, respectively. AUC0- ∞ for DOX and doxobubicinol without enalapril was 1167.73 (45.26) hr*ng/ml and 1056.32 (92.03) hr*ng/ml, respectively. There is no interaction between DOX and enalapril. Enalapril was tolerated (33% grade 1 dizziness).

**Conclusion:**

ACEI, enalapril, does not appear to alter the PK of DOX. Ongoing efforts to determine the effectiveness of ACEI as a cardioprotective agent in women receiving DOX chemotherapy should be continued.

## Introduction

Doxorubicin is an anthracycline chemotherapeutic agent that is the backbone of standard curative-intent chemotherapy for stage 1–3 breast cancer (Lyman 2010; Gianni et al. 2009). While the immediate side effects of doxorubicin such as myelosuppression, nausea, and vomiting are reversible, doxorubicin is associated with dose-related cardiotoxicity, including cardiomyopathy and congestive heart failure that is irreversible (Swain 1999; Bird and Swain 2008; Lenihan and Cardinale 2012). Symptomatic heart failure can occur in 3-4% of patients receiving cumulative doses of 400–500 mg/m^2^ and more than 30% in patients receiving ≥ 600 mg/m^2^ (Singal and Iliskovic 1998; Yeh et al. 2004; Muggia and Speyer 1999). Asymptomatic declines in ejection fraction occur in up to 20-25% of patients treated with moderate doses of doxorubicin (i.e. 240–400 mg/m^2^) and up to 30-35% of patients treated with higher doses (Lenihan and Cardinale 2012). This cardiac toxicity can occur acutely or several years later.

Given the importance of anthracyclines in treating breast cancer, various strategies have been tried to prevent or ameliorate the cardiac toxicity associated with doxorubicin including the use of concurrent medications like angiotensin converting enzyme inhibitors (ACEI) (Cardinale et al. 2006; Bosch et al. 2013; Georgakopoulos et al. 2010), beta-blockers (Kalay et al. 2006), dexrazoxane (Swain et al. 1997), liposomal formulations of doxorubicin chemotherapy, or the alteration of doxorubicin infusion times (Blaes 2010). In animal models, the use of ACEI with doxorubicin has been shown to ameliorate the cardiac toxicity (Ibrahim et al. 2009). In retrospective studies, concomitant use of ACEI appears to help prevent cardiac toxicity (Blaes et al. 2010). In prospective studies, the use of ACEI in patients who have had an elevation in troponin-I after chemotherapy also appeared protective as secondary prevention (Bosch et al. 2013; Georgakopoulos et al. 2010). Cardinale et al. evaluated 114 patients who received high dose chemotherapy (Cardinale et al. 2006). At 12 months after therapy, the patients with an elevation in troponin T randomized to enalapril 20 mg daily had better left ventricular ejection fraction (62.8% vs 48.3%, p < 0.001) as compared to those on a placebo. A subsequent study demonstrated that patients with non-Hodgkin lymphoma treated with anthracycline based chemotherapy who received an angiotensin II receptor blocker, a medication that also works on the renin-angiotensin system, had no transient changes in left ventricular end diastolic diameter as compared to those not treated with an angiotensin II receptor blocker (Nakamae et al. 2005). While the exact mechanism of how ACEI may help ameliorate doxorubicin cardiac toxicity is unclear, it is hypothesized that ACEI may attenuate the peroxidizing action of doxorubicin and affect nitrous oxide production, thus reducing cardiac toxicity (Iqbal et al. 2008). It is unclear whether some of ACEI effects are based on changes in hemodynamics.

Despite the encouraging data that ACEI and other medications working on the renin-angiotenin system may prevent doxorubicin cardiac toxicity, questions remain as to whether the concomitant medication use will alter the efficacy of doxorubicin. Doxorubicin is metabolized to doxorubicinol by ubiquitous aldoketoreductase enzymes (Piscitelli et al. 1993; Benjamin et al. 1973). These aldoreductase enzymes subsequently have a number of downstream pathways that affect cell growth and proliferation. These enzymes are not typically inhibited or induced by other drugs. Concurrent ACEI such as enalapril, however, may reduce the conversion of doxorubicin to its active metabolite, doxorubicinol, thereby preventing cardiac toxicity but also reducing anticancer efficacy. Given the lack of data to support enalapril as an inhibitor of the major enzymes involved in doxorubicin metabolism, the potential for an interaction is low. However, epidemiologic studies have reported conflicting reports as to whether the use of ACEI in those receiving chemotherapy alters outcomes. Ganz et al. reported there was an increase in the risk of recurrence in patients taking ACEI the year before and after a breast cancer diagnosis (HR 1.56) (Ganz et al. 2011). This data was refuted by Chae et al. who reported that there was a decreased risk of recurrence in those treated with ACEI with or after a breast cancer diagnosis (HR 0.60) (Chae et al. 2011), as well as by an analysis with the Danish cooperative group data registry (Sorensen et al. 2013). While not all of these subjects received doxorubicin, it is unclear whether ACEI use alters outcomes.

In order to proceed to a cardioprotection clinical trial, the lack of an interaction between doxorubicin and ACEI enalapril needs confirmation. This paper reports the results of a randomized, cross-over, single blinded drug drug interaction study to evaluate whether ACEI enalapril affects systemic doxorubicin and doxorubicinol exposure.

## Methods

### Subjects and methods

Nonpregnant women over the age of 18 years with stage 1–3 breast cancer prescribed doxorubicin and cyclophosphamide every 14 days (dose dense AC) for four cycles were eligible for enrollment. Normal liver and kidney function were required. Subjects with a history of cardiovascular disease or a diagnosis of hypertension were excluded. Subjects with active use of an angiotensin-converting enzyme inhibitor, use of an angiotensin receptor blocker or a known allergy to enalapril were not eligible to participate. Subjects known to be taking any cytochrome P450 inducers or inhibitors (Table 1) were not eligible. The exception to this was the anti-emetic and CYP inducer aprepitant (Shadle et al. 2004), which was administered to all subjects. Herbal supplements were not allowed while on the study or the week prior to receiving doxorubicin. All subjects furthermore agreed to not consume grapefruit juice while on the study.

**Table 1:** **Cytochrome P450 inhibitors and inducers**

***3A4, 5, 7 Inhibitors:***	***3A4, 5, 7 Inducers***
Indinavir	Carbamazepime
Nelfinavir	Phenobarbital
Ritonavir	Phenytoin
Amiodarone	Rifabutin
Azithromycin	Rifampin
Cimetidine	St. John Wort
Clarithromycin	Troglitazone
Diltiazem	Aprepitant*
Erythromycin	
Fluvoxamine	
Grapefruit Juice	
Itraconazole	
Ketoconazole	
Mibefradil	
Nefazodone	
Troleandomycin	
Verapami	

*All subjects in study used aprepitant.

The protocol and analysis were approved by the University of Minnesota Institutional Review Board and Cancer Center Review Committee. All patients provided written informed consent according to the Declaration of Helsinki. The clinical trial was registered at ClinicalTrials.gov (NCT00895414).

Doxorubicin (60 mg/m^2^) was administered as an IV infusion over 5–10 minutes followed by an infusion of cyclophosphamide 600 mg/m^2^ over 30–60 minutes every two weeks for a total of four cycles (cumulative doxorubicin 240 mg/m^2^). All subjects received aprepitant, dexamethasone and palonsetron prior to each infusion of doxorubicin. All subjects received pegfilgrastim subcutaneously 24–72 hours after doxorubicin and cyclophosphamide.

This was a randomized, cross-over, single-blinded drug-drug interaction study. All patients received one cycle of AC chemotherapy with enalapril and another cycle of AC with placebo (without enalapril). The two study cycles were consecutive. Patients were randomized to the enalapril intervention or placebo in cycle 1 with cross-over in the subsequent cycle. They began 5 mg of enalapril daily on day −6, and then increased to 10 mg daily on day −2, prior to doxorubicin administration on day 1.

Blood samples for pharmacokinetics (doxorubicin and doxorubicinol) were obtained in each subject twice (one cycle with enalapril and one with placebo). Samples were drawn at baseline (immediately prior to start of doxorubicin) and then at 0.5, 1, 2, 4, 24 and 48 hours after the end of infusion. Five milliliters of blood was collected at each sampling time and placed in an EDTA tube for doxorubicin and doxorubicinol analysis. Each sample of whole blood was immediately inverted 10 times, placed on wet ice, centrifuged at 3400 rpm for 10 minutes at 4 degrees C, plasma separated and frozen to −80 degrees C within 15 minutes of collection. Samples remained frozen at −80 degrees C until time of analysis. Samples were batched at the time of analysis.

### Pharmacokinetic analysis and bioanalytical methods

Doxorubicin and doxorubicinol plasma concentration-time data were analyzed using noncompartmental methods (WinNonLin Professional 6.3). Area-under-the-curve (AUC) _(0-∞)_ was estimated by the log/linear trapezoidal method as AUC _(0-t*)_ + C(t*)/Ke where C(t*) was the last observed concentration and Ke is the terminal first order elimination rate constant. Ke was calculated as the slope of the linear portion of the log of plasma-concentration vs time curve using linear regression analysis. C_max_ was at the highest observed concentration.

### Doxorubicin & doxorubicinol assay

Detection and quantification of doxorubicin and doxorubicinol in plasma was performed using high-performance liquid chromatography (Agilent 1200 Series, Santa Clara CA) coupled with a TSQ Quantum triple stage quadrupole mass spectrometer (Thermo-Electron, San Jose, CA) using a previously published method with minor modifications (DiFrancesco et al. 2007). The chromatographic separation was performed with a Waters UPLC BEH C18, 2.1 × 50 mm, reversed phase column with a 1.7-micron particle size (Waters, Milford, MA). The mobile phase used for gradient elution consisted of (A) 5 mM ammonium acetate in water, pH 3.5 (B) methanol. The gradient was linear from 45-95% (B) in 4 min, at a flow rate of 0.1 mL/min, for a total run time of 8.5 minutes. The column temperature was maintained at 35°C. The detector settings of the TSQ Quantum were an ESI with the stainless steel spray needle, positive polarity ionization, selective reaction monitoring mode (SRM); spray voltage, 4500 V; capillary temperature, 400°C; argon collision gas pressure, 1.5 mTorr; unit resolution for Q1 and Q3, 0.7 u (FWHM); and ions detected (m/z), daunorubicin (internal standard) precursor 546, product 363; doxorubicin precursor 544, product 397 and doxorubicinol precursor 546, product 363. The collision energy was 14 eV, 12 eV and 25 eV, respectively. Following the addition of the internal standard (125 ng of daunorubicin) and 0.1 M HCl (0.25 mL), EDTA plasma samples (0.25 mL) were extracted using a Supel-Select HLB SPE cartridge (30 mg/1 mL; Sigma-Aldrich, St. Louis, MO). Cartridges were conditioned with 1 mL methanol followed by 1 mL DI water (18 Mohm.cm, type 1). The cartridges were loaded with the samples and centrifuged at 83 × g for 5 minutes and then washed with 1 mL distilled ionized water, centrifuged and transferred to a vacuum manifold for 5 minutes, and eluted with 1 mL methanol. The eluent was evaporated to dryness using a nitrogen evaporator (Zymark Turbo Vap LV, Hopkinton, MA) set at 37°C, and reconstituted in 100 μL of mobile phase (A:B, 45:55). Doxorubicin and doxorubicinol were obtained from Toronto Research Chemicals (Toronto, Ontario, CAN). Daunorubicin was obtained from Sigma-Aldrich (St. Louis, MO). The assays are linear in the range of 1 – 1,000 ng/mL. Doxorubicin accuracy and total variability was 102% and 6.6%, respectively. Doxorubicinol accuracy and total variability was 103% and 10.5%, respectively. The lower limit of quantification for both analytes is 1 ng/mL.

### Statistics

The primary objective was to demonstrate that enalapril use did not result in an increase or decrease in doxorubicin. The 90% confidence intervals around the geometric mean ratios of the pharmacokinetic measures (doxorubicin and doxorubicinol AUC_(0-∞)_ and C_max_) with and without enalapril were calculated. The confidence interval is a measure of the precision of the exposure ratio estimate. Confidence interval bounds within 80-125% were considered evidence of no drug-drug interaction (U.S. Department of Health and Human Services, Food and Drug Administration, Center for Drug Evaluation and Research (CDER) 2012).

## Results

### Demographics

Nineteen women with a median age of 47 (range 28–68) years with no cardiac history were enrolled. Baseline characteristics of the enrolled women are provided in Table 2. Median body mass index was 25.3 kg/m^2^ (range 21.5-53.0). Median ejection fraction (EF) prior to start of therapy was 61% (range 48-75%). Kidney and liver function were normal in all subjects. One woman withdrew from the study due to pulmonary embolus, and another woman had pharmacokinetic laboratories drawn incorrectly; therefore final pharmacokinetic analyses were performed on seventeen women.

**Table 2:** **Baseline characteristics of subjects (n = 19)**

	**Mean**	**SD**	**Min**	**Max**
Age (years)	44.5	11.1	28.0	68.0
Baseline ejection fraction (%)	62.1	6.0	48.0	75.0
Height (centimeters)	164.9	8.2	152.0	188.0
Actual weight (kilograms)	75.1	21.6	54.5	140.4
BMI (kilograms/meters^2^)	28.2	8.2	21.5	53.0
BSA (mg/m^2^)	1.9	0.3	1.6	2.5
Aspartate transaminase (AST) (IU/L)	26.2	4.0	18.0	33.0
Alanine transaminase (ALT) (IU/L)	27.2	12.1	8.0	62.0
Total bilirubin (mg/dL)	0.6	0.2	0.2	1.0
Creatinine (g/dL)	0.7	0.1	0.5	1.0

### Pharmacokinetics

**Figures 1:**
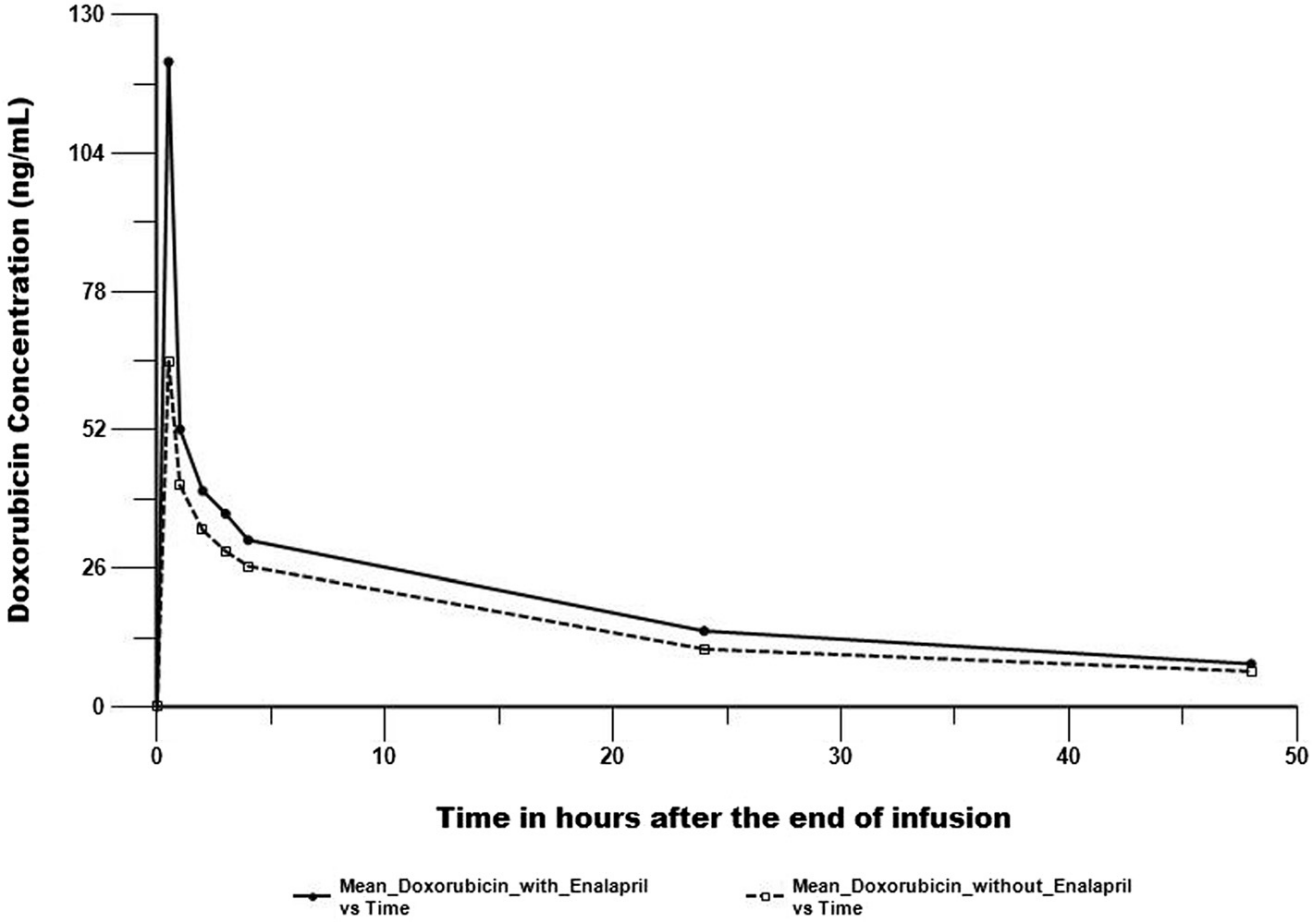
**Pharmacokinetic plots of doxorubicin.**

**Figures 2:**
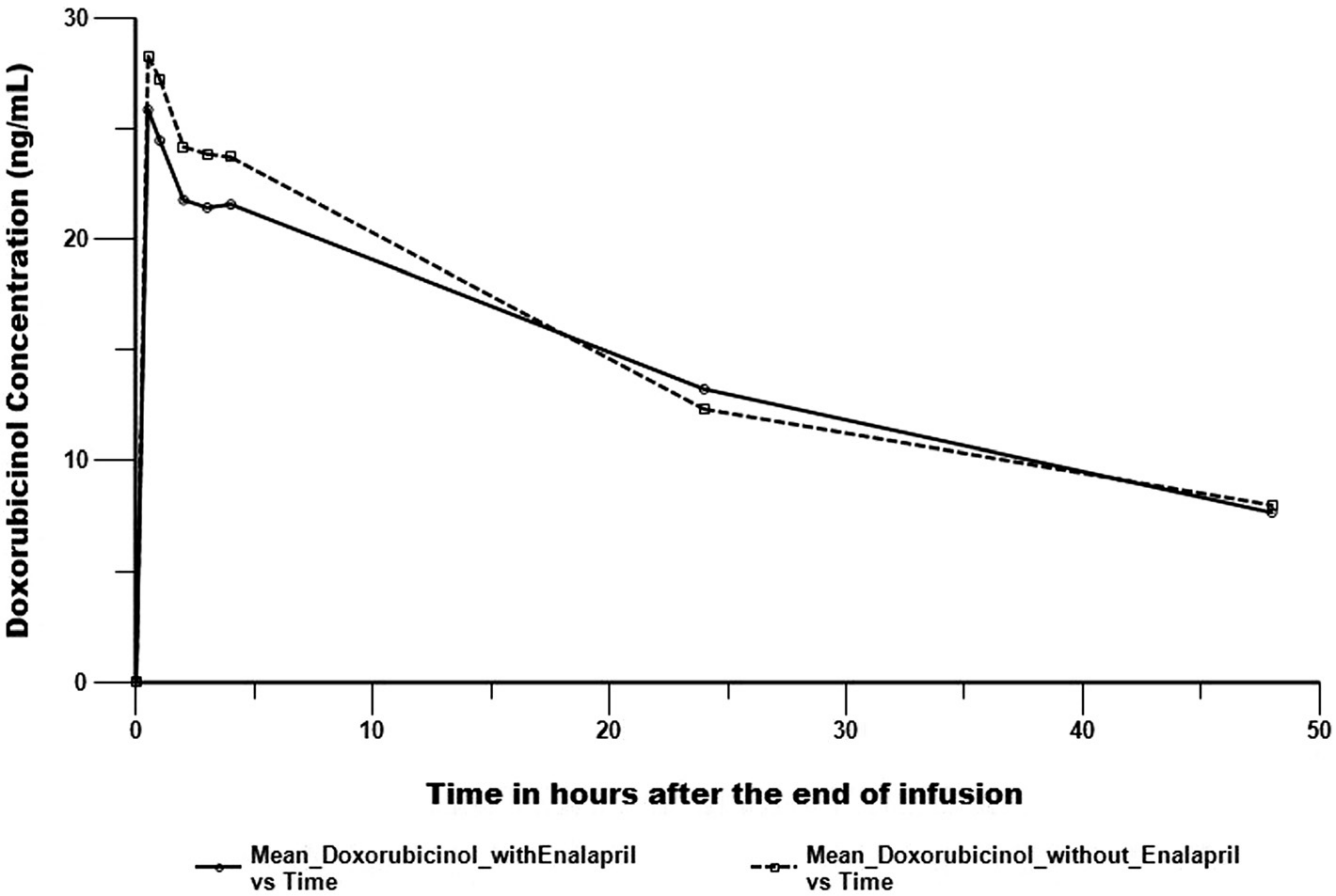
**Pharmacokinetic plots of doxorubicinol.**

Plasma pharmacokinetic profiles of doxorubicin and its metabolite doxorubicinol are shown in Figures 1 and 2. Mean (standard error) AUC_(0-∞)_ for doxorubicin and doxorubicinol with enalapril exposure was 1185.6 (44.6) ng-hr/mL and 1040.0 (80.6) ng-hr/mL, respectively. AUC_(0-∞)_ for doxorubicin and doxorubicinol without enalapril was 1167.73 (45.3) ng-hr/mL and 1056.3 (92.0) ng-hr/mL, respectively (Table 3). Doxorubicin AUC_(0-∞)_ and Cmax geometric mean ratios (90% CI) were 1.02(0.96-1.08) and 1.19(0.94-1.51), respectively.

**Table 3:** **Doxorubicin and doxorubicinol pharmacokinetics (geometric mean+/−standard error)**

	**Doxorubicin**		**Doxorubicinol**	
	**With enalapril**	**Without enalapril**	**With enalapril**	**Without enalapril**
Cmax (ng/mL)	88.79 ± 0.30	79.96 ± 5.16	27.81 ± 3.59	29.66 ± 4.68
AUC_(0-∞)_ ng-hr/mL	1185.56 ± 44.64	1167.73 ± 45.26	1040 ± 80.6	1056.32 ± 92.03

The Cmax 90% CI exceeded the upper boundary of 125%. Doxorubicinol AUC_(0-∞)_ and Cmax geometric mean ratios (90% CI) were 0.96(0.88-1.05) and 0.99(0.91-1.08), respectively with all 90% CIs falling within the acceptable boundaries (80-125%). Because the doxorubicin Cmax upper boundary was exceeded, the analyses were reevaluated eliminating the subject with a body mass index of 53 kg/m^2^. Literature suggests obese patients typically have a prolonged elimination half-life which may not affect Cmax concentrations (Rodvold et al. 1988; Hanley et al. 2010). In this subject while receiving enalapril, the doxorubicin Cmax was 638.91 ng/ml which is nearly ten-fold higher than without enalapril where Cmax was 77.89 ng/ml; the AUCs_(0-∞),_ however_,_ were not significantly different between the study periods (1642.05 ng-hr/ml with enalapril compared to 1296.39 ng-hr/ml without enalapril). In reanalysis removing the outlier Cmax, the 90% CI for Cmax was 0.94-1.21 and within the acceptable boundaries. As a result, it appears the Cmax value may have been obtained from a line with residual doxorubicin and does not reflect a true Cmax.

### Safety

There were no serious or grade 3 or 4 adverse events. The most commonly reported drug-related adverse event was dizziness with 33% experiencing grade 1 dizziness. No clinical cardiac events occurred.

## Discussion

In our study, the addition of ACEI enalapril to doxorubicin had no effect on doxorubicin or doxorubicinol pharmacokinetics. The geometric mean AUC ratios of doxorubicin and doxorubicinol showed no significant differences while exposed or unexposed to ACEI enalapril. While the sample size is small, our study was adequately powered to determine whether enalapril affected the pharmacokinetics of doxorubicin. No interaction was observed.

While some use of anthracyclines has declined over time, doxorubicin continues to be the backbone of curative intent chemotherapy in many solid tumors including breast cancer. Doxorubicin in combination with other chemotherapy agents has been shown to reduce the recurrence of breast cancer by approximately one-third (Gianni et al. 2009). Some investigators, however, have suggested not using anthracycline-based chemotherapy in the adjuvant treatment of stage 1–3 breast cancer due to the cardiac concerns (Jones and Ewer 2006). Jones et al. published a non-inferiority study looking at the use of docetaxel and cyclophosphamide (TC) as compared with doxorubicin and cyclophosphamide (AC) (Jones et al. 2009). In other trials such as the Breast Cancer International Research Group 006, however, longer follow-up demonstrated there were 20% more recurrences and death with a non-anthracycline-containing regimen consisting of docetaxel, carboplatin and trastuzumab (TCH) compared with the anthracycline containing regimen (AC followed by paclitaxel and trastuzumab TH) (Lyman 2010; Gianni et al. 2009; Slamon et al. 2011). The greatest benefit was demonstrated in those with early stage, node negative disease. As a result, anthracyclines will continue to play a role in the treatment of early stage breast cancer, and they continue to be used in contemporary clinical trials (NCT01042379, NCT01966471). Limiting the cardiac toxicity of the doxorubicin in use continues to be important.

Out study did not demonstrate an interaction between ACEI enalapril and doxorubicin or its metabolites. Our study, however, does not explain whether enalapril affects cellular interactions or intracellular chemotherapy concentrations. These cellular interactions may explain the observed differences in the epidemiologic studies (Ganz et al. 2011). It is possible that ACEI enalapril acts as a free radical scavenger of various reactive oxygen species; as a result, ACEI may alter the effects of doxorubicin through antioxidant properties (Ibrahim et al. 2009), but not through pharmacokinetic changes.

In conclusion, no differences were observed in pharmacokinetic measurements when enalapril is administered with doxorubicin. These data demonstrate that no dose adjustments in doxorubicin are required when enalapril is co-administered. Based on the results of our work, we suggest that further work into the cardiac preventive effects of enalapril is warranted.
